# Prioritizing Career Preparation: Learning Achievements and Extracurricular Activities of Undergraduate Students for Future Success

**DOI:** 10.3390/bs13070611

**Published:** 2023-07-24

**Authors:** Dongsuk Kang

**Affiliations:** Department of Business Administration, College of Social Sciences, Gangneung-Wonju National University (GWNU), Street 7, Jukheon-gil, Gangneung-si 25457, Republic of Korea; professional@gwnu.ac.kr

**Keywords:** job preparation, career development, occupation seeking, university management, educational innovation

## Abstract

Preparing for a job can be difficult for undergraduates as this would be one of their first experiences of responsibility; obtaining a job will make them economically independent beings taking responsibility for their lives. Since the COVID-19 pandemic, this task has become even more challenging for Generation Z students, born in the mid-1990s, as they navigate a turbulent job market. This study aims to analyze undergraduates’ priority decisions regarding the criteria and activities of their career preparation. The study conducted a questionnaire analysis using the methodology of analytic hierarchy process (AHP) with 93 university students in the Republic of Korea. This research finds that students rank personal feelings of achievement as the most important criterion in their career preparation. They perceive extracurricular activities and internships as the most beneficial experiences for job readiness. On the contrary, networking activities within the university and with alumni received the least importance. These results highlight a need for universities to innovate their educational approach. Addressing the gap between current curricula and student needs and enhancing self-efficacy among students are critical. Innovative educational strategies could be a key to meeting societal expectations, such as the integration of business and technology, and catering to the unique learning needs of Generation Z. This becomes particularly relevant considering the rise of new career paths, such as youth startups, leveraging advanced technologies.

## 1. Introduction

The COVID-19 pandemic, along with resulting policies such as social distancing and travel restrictions, led to a global economic standstill for over two years. In the Republic of Korea (Hereinafter referred to as Korea), university students and graduates seeking jobs have faced decreased employment opportunities and increased anxiety due to significant changes in companies’ hiring processes. For instance, there has been a shift from hiring new graduates to experienced workers, and the use of artificial intelligence in job interviews has become more common [1,2]. As a result, many students are opting to prepare for public service exams, drawn by the reliable employment information, law-guaranteed retirement age of 60, and predictable job tasks [1].

**Figure 1:**
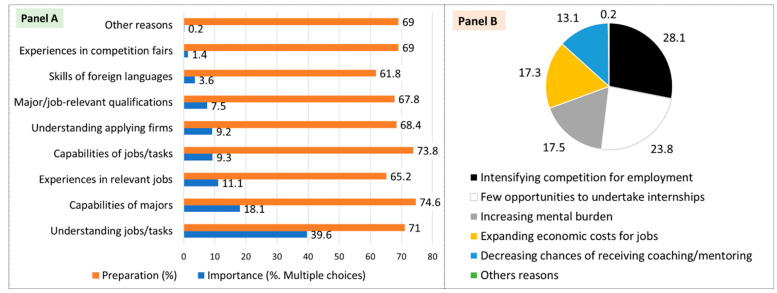
Korean undergraduates’ thoughts on their preparation level and important factors on job preparation. Notes. All measurement units are expressed as percentages (%). Importance (**Panel A**) and difficulties (**Panel B**) are the result of multiple choices. The sample size was 6006 undergraduate students in Korea. Source: Kim, Min [1], designed and integrated by the author.

The employment rate for university graduates aged 25–34 in Korea stands at 75.2% as of 2020, ranking 31st among the 37 OECD member countries [3]. Moreover, the discrepancy between graduates’ university majors and their occupations is at 50%—a rate higher than 22 other OECD member countries [3]. A survey conducted in December 2021 showed that Korean undergraduates prioritize understanding their desired jobs (39.6%), improving their major competency (18.1%), and gaining related experience (11.1%) in their job preparation (Panel A in Figure 1).

However, their preparation level for careers was generally low at 61.8–74.6%, and the skills of foreign language and performing an internship were also low (Panel B in Figure 1). Their main difficulties in preparing for employment were intensifying competition for jobs (28.1%), decreasing/lacking opportunities for work experience (e.g., internships: 23.8%), and an increasing psychological burden for developing careers (17.5%; Panel B in Figure 1).

Previous research has identified several influential factors for students’ future employment, utilizing methodologies such as analytic hierarchy processes (AHP [4,5,6]) and structural equation modeling (SEM) of the questionnaire analysis [7,8,9,10]. While some studies have highlighted useful evaluative criteria (e.g., importance and performance [11,12]) and job preparation strategies [4], there seems to be a lack of an integrated framework that encapsulates students’ perceptions and practical career development options. The surprising events of COVID-19 have drastically changed the socioeconomic environments of universities and students [1,2,13,14].

Therefore, this study aims to analyze the main criteria and alternative activities for undergraduates’ employment preparation. This study raises **two research questions (RQ):** Which criterion do students evaluate as the most meaningful for their careers (**RQ1**)? Which activity do they rate as most significant for employment preparation (**RQ2**)? 

The results of AHP with a sample of 93 Korean undergraduates showed that their feeling of achievement was the most meaningful criterion. Extracurricular activities (e.g., participating in competition fairs) and internships were evaluated as the most valuable alternatives for job preparation. These findings could offer insights to students and universities in preparation for developing the students’ careers. Students’ desire for extracurricular activities and internships could be a missing element on which the existing university education does not focus sufficiently.

This study could highlight that higher education must modernize its curriculum to better prepare students for the rapidly evolving job market, which includes new careers such as YouTubers and youth startups. This is particularly important in light of the fourth industrial revolution, characterized by the convergence of technologies such as hyperscale artificial intelligence and ChatGPT, blockchain, cryptocurrency, non-fungible tokens (NFTs), and the metaverse. The study highlights the need for a more practical, hands-on approach to learning in higher education, especially in Korea’s unique export-driven environment [15].

## 2. Literature Review

### 2.1. Criteria for Career Preparation: Importance, Performance, and External Assistance

The preparation of undergraduates for employment could involve thinking about their expected roles at work, so that they take responsibility for their lives economically while practically adapting to the new social environment beyond their sheltered university [7]. Some factors that consumers of their labor (e.g., firms, public organizations) may consider when recruiting could be particularly important from the viewpoint of students obtaining their preferred jobs. In particular, the criterion of judgment for job seeking could include (relative) importance and performance, which has been adopted as a major component of importance–performance analysis (IPA) [11,12,16].

IPA can be applied to decision making with various multi-criteria [11], and it has been considered a useful tool in survey analysis with interval measurements (e.g., five- or seven-point Likert scale) to evaluate customers’ satisfaction with a product or service [12,16]. It has also been utilized for performance/policy evaluation in the public sector (e.g., universities and government services) as well as in private companies [17,18]. Researchers have adopted IPA in conjunction with strengths–weaknesses–opportunities–threats (SWOT), which can intuitively analyze an organization’s competitive forces and environmental factors [11,12].

From the traditional viewpoint of IPA, importance can denote the perceived meaning of a customer’s purchasing experience in a service or product purchase experience, and performance could be relevant to the level of achievement that they realize through the experience [11,16]. While these two criteria could be somewhat independent of each other in the sense of their measurement, they need to be interconnected so that the total level of the service/product improves with an increase in both importance and performance [12].

Therefore, this study utilizes importance and performance as representative judgment criteria in the efforts of undergraduates to prepare for employment. This study adopts Azzopardi and Nash [11]’s definition of the two criteria, defining importance as a relative significance or influence when students prioritize their preparation activities for jobs and defining performance as the degree of achievement or effort in preparing for future employment.

Importance and performance could be linked to concepts of concern and confidence in career adaptability, which is one’s psychological resource or ability to perform tasks expected from vocational roles [8]. Concern can refer to one’s value or meaning in their career-related future [9,10], and the increase in concern could be the high value of the importance of job preparation activities. Confidence can denote a feeling of being focused on something in order to achieve one’s goals or desires for success [7,9,10], and a high value of confidence can be connected to one’s level of performance/achievement.

On the other hand, it could be critical for students to distinguish between the job-preparation factors which they could achieve themselves and those that they could achieve with others’ help. IPA traditionally evaluates competitive capabilities by establishing four quadrants with two axes, namely, importance and performance [11,12]. This approach may tacitly assume that one can check and improve one’s competitive edge without external support; however, it could be important for undergraduates to receive help/mentoring through interacting/networking with universities, employment-related institutions, and/or acquaintances during their early career development to develop career competency [7]. Therefore, this study includes the importance, achievement, and need for external help from others as meaningful criteria for undergraduates’ preparation for future jobs.

### 2.2. Integration with IPA and AHP, and AHP Applications for Career Preparation

IPA can be applied to integrated analysis with complementary methodologies of AHP and other multi-criteria decision-making methods (MCDM). For example, Hongshan Zoo, a famous tourist destination in China, was evaluated using the combined analytic framework of IPA and AHP to measure various aspects of attractiveness and competitive positioning [19]. The integrated analysis of IPA, linear regression, and decision-making trial and evaluation laboratory (DEMATEL) was adopted to rate the quality levels of suppliers in Taiwan’s computer industry [16]. IPA and cluster analysis were used to evaluate the bus services in Tehran, Iran [17].

Some research has utilized AHP to determine the priorities and value weights of student preparation and influential factors for future careers. The AHP was adopted to evaluate the priority of influential variables in the selection of majors by medical students in Taiwan [4] and rate the major factors in the employment of female undergraduates in the UAE [5]. It was also used to prioritize factors of career and professional skills in choosing a nursing major for Turkish high school students and parents [6].

### 2.3. Alternative Activities for Job Preparation

Alternative activities for undergraduates’ career preparation could be grade point average (GPA) [1,7] and/or understanding of their majors [6], networking with alumni and the university’s reputation [6,20], internships or other relevant career experiences [1,21], employment-related qualifications [1], and extracurricular activities of participating in competition fairs/exhibitions [1]. Achievements in one’s major (e.g., high level of GPA) is one of the basic and quantitative factors that employers can use to evaluate the applicants’ performance as their potential competency in major-related vocations [7]. Students’ utilization (networking) of their university reputation/brand could be relevant to the distinctive image of outstanding alumni and of the university from an employer’s perspective [22]. It has also been evaluated as one of the important indices that Quacquarelli Symonds (QS), Times Higher Education (THE), and other evaluators of world universities all incorporate in their measurements [20].

Internship and experiences related to one’s preferred job could be plausible signals for finding a job. One’s career of good internship with high skills could contribute to future employment and improve employment awareness [21]. External activities such as acquiring employment-related qualifications/licenses and participating in competition fairs/exhibitions could also be influential factors for careers, indicating that Korean undergraduates have been consciously preparing individually and/or as a team [1].

## 3. Research Model and Methodology of AHP

### 3.1. Research Model

**Figure 2:**
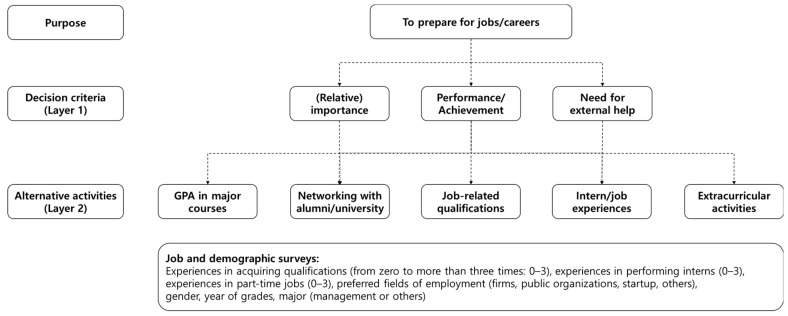
The research model of this study.

This study proposes a research model (Figure 2) that consists of three criteria for judgment and alternative activities for the career/job preparation of undergraduates by reviewing and integrating relevant research (Table 1).

### 3.2. Statistical Method: AHP

This study utilizes the AHP, which is one of the representative MCDM that can evaluate diverse options by interlinking/comparing goals, criteria, and alternatives. It is a type of simplified version of the analytic network process (ANP) [23,24]. It can rate not only the relative value (weights) of alternatives according to each criterion but also the sub-factors of elements (e.g., the comparison between SWOT/SWOC and the evaluation of their components [25,26]).

Researchers must consider two representative factors (i.e., the logical structure of the layers and elements in the layer and the appropriate number of elements in the layer) when conducting a research model using AHP. Above all, the logicality of the layers and elements could be the fulfillment of the mutually exclusive and collective exhaustiveness (MECE) of the elements in the layer and the layers for reasonable decision making. Moreover, the number of elements in the layer could be relevant to the comparison burden of respondents and the preservation of the consistency level in the pairwise comparison between elements. In this study, AHP performs a pairwise comparison between elements of the first layer (three criteria) and between elements of the second layer (five alternatives) according to the elements of the first layer (Figure 2).

**Figure 3:**
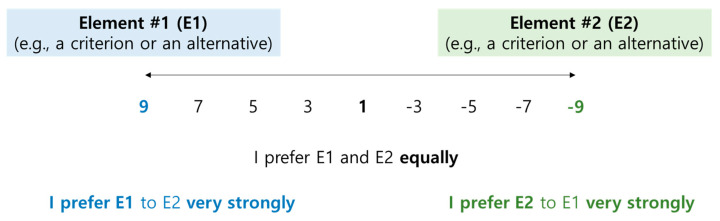
Nine degree of answer points in respondents’ pairwise comparison. Notes. This research revises Saaty [23], Podvezko [28], and Zhou and Du [29]’s expressions.

If the number of elements in a layer increases, the number of questions that respondents should answer (i.e., the size of the comparison matrix) also increases [24]. For example, the number range of elements (e.g., 7±2) could be appropriate for respondents to compare because if the number is higher than seven, the increase in the value of random index (*RI*) becomes very small, and respondents may find it difficult to compare elements [27]. In addition, the increase in the number of elements and/or the degree of comparison scales (e.g., 1, 2, …, 9) could lead to the respondents’ burden to compare elements and the inconsistency of their comparison results [28]. Therefore, this study adopted a five-point comparison scale (that is, 1, 3, 5, 7, and 9. [28]) to compare the criteria and alternatives (Figure 3).

**Figure 4:**
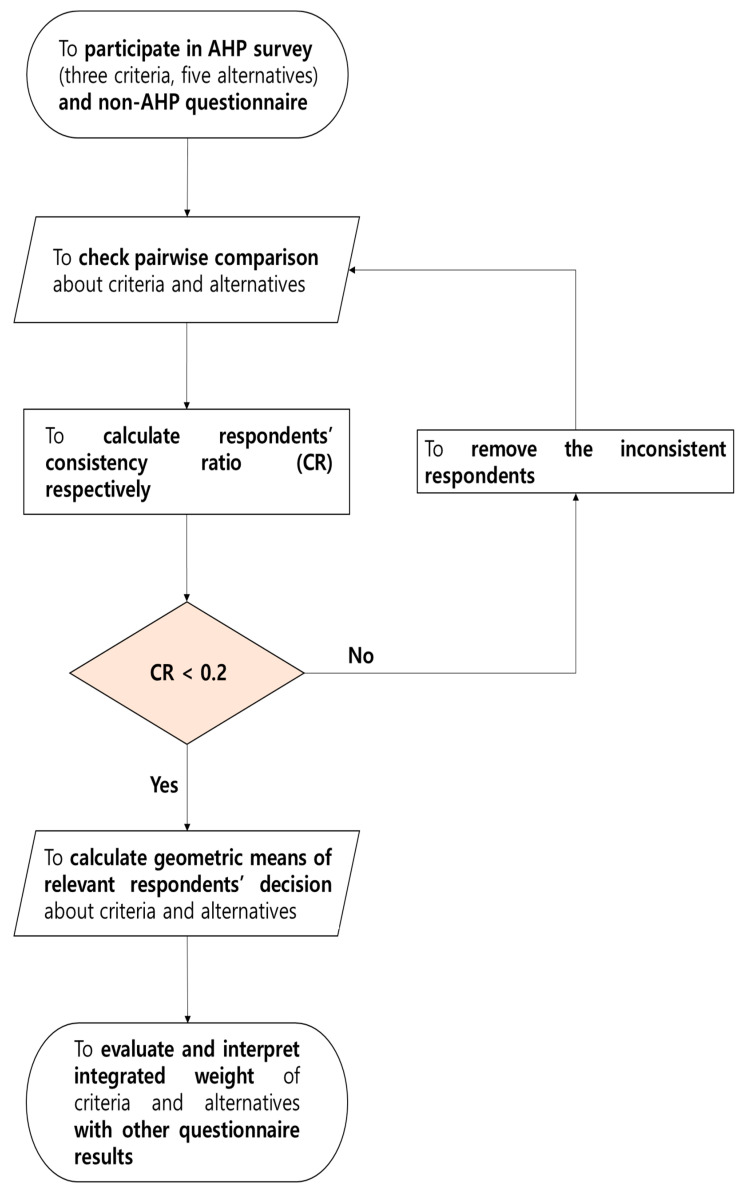
Analytic flow of this research. Notes. This study adopts and revises the analytic framework of Byun [30] and Yoon and Choi [31].

The AHP questionnaire was designed in accordance with the research model (Figure 2) and the analytic flow (Figure 4). This study established 33 AHP-related questions ($=\left(\begin{array}{c} 3\\ 2 \end{array}\right)+3\times \left(\begin{array}{c} 5\\ 2 \end{array}\right)$ = 3 + 3×10) and eight demographic questions (Table 2). After designing the questionnaire and implementing the survey, the study drew the value of consistency ratio (*CR*) by calculating the values of the pairwise comparison between criteria and alternatives. For comparison results in which the *CR* values of respondents were lower than 0.2, the study established the weights of criteria and alternatives by using geometric means to synthesize the results.

Researchers can consider two main methods for integrating respondents’ results (group AHP): a geometric mean of the comparison results that derives the priority result by synthesizing the respondents’ respective results comparing individual elements; and a weighted arithmetic mean that synthesizes the respondents’ priority results after their comparison of elements [24]. The geometric mean could be meaningful as a methodology to preserve the reciprocal property of the results on the pairwise comparison of elements [24].

**Figure 5:**
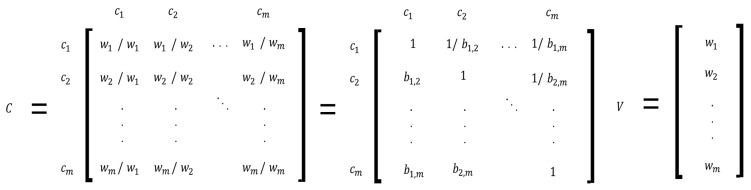
The matrix of pairwise comparison in the element $C_{}^{}$. Notes. This research revises Saaty [23], Zhou and Du [29], Kubler, and Robert [32]’s expressions. Elements ($C_{1}^{},\;C_{2}^{},\ldots ,\;C_{m}^{}$ with value $v_{i,j}^{}.\;i,j=1,\;2,\;\ldots ,\;m$) in a layer *k*. $b_{i,j}^{}=w_{j}^{}/w_{i}^{}.$ $w_{i}^{}:$ a respondent *l*’s decision weights on element *i*. $b_{i,j}^{}$ is measured by a respondent *l*’s answer to the question about the pairwise comparison between element *i* and *j* (please see Figure 2).

**Figure 6:**
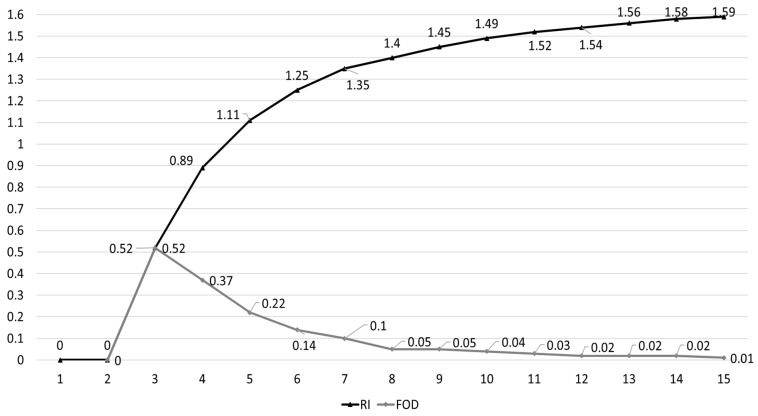
Values of random inconsistency with the number of elements. Notes. This research adopts Saaty [23] and Zhou and Du [29]’s values of RI. RI: random inconsistency or index; FOD: first-order differences.

AHP can calculate the weight of each element through a pairwise comparison between elements (e.g., criteria and alternatives) within the hierarchy (Figure 5). Researchers can draw the value of *CR* to determine the consistency of the respondents’ pairwise comparison results (see Equations (1)–(3) and Figure 6).

(1) $$Cv=mV\;\mathrm{or}\;\left(C-mI\right)V=0.\;C^{\prime }V^{\prime }=\lambda_{max}^{}V’.$$

(2) CI=(λmax−m)(m−1).

(3) CR=CI/RI.

Notes. This research utilizes and revises Saaty [23]’s expression. C: comparison matrix of a choice/element set (Figure 2); V: matrix of values or importance on choices; m: number of choices; $C^{\prime }$: transpose (or judgement) matrix of *C*; $\lambda_{max}^{}$: maximum eigenvalue of the matrix of judgement/choice-comparison; CR: consistency ratio; CI: consistency index; RI: random index. The value of RI could be determined by the matrix size, with an average of 50,000 computations [23]. The above equations are identically applicable to the AHP and ANP methods [23].

Reciprocity (e.g., $b_{i,k}^{}=b_{i,j}^{-1}$ (please see Figure 5)) and transitivity (e.g., $b_{i,k}^{}=b_{i,j}^{}\times b_{j,k}^{}.\;i\neq j\neq k$.) must be preserved to be consistent with the comparison result [24,32]. Researchers can check the fulfillment of these conditions by comparing *CR* with some standard values (e.g., *CR* < 0.1 [23]). Random index (RI in Equation (3)) shows the probability value of a matrix composed of random numbers generated through simulation. The value (*CR* = 0.1) could denote that the probability of randomness is 10% in the pairwise comparison of the respondents’ judgment [30].

The *CR* value is a criterion for ensuring the consistency of results between element comparisons (e.g., transitivity and reciprocity of comparison results), but it is not a standard for evaluating the quality/professionalism of respondents [24]. If the *CR* is higher than the standard value, researchers need to request the survey participants to revise their answers or to check the most inconsistent decision factor(s) and modify the unreliable value(s) of the factor(s) [33].

However, conducting a repetitive survey to adjust the respondents’ judgment [33] may cause practical issues, such as their rejection/burden of revising judgment and costs of re-surveying [30,31]. Moreover, the standard value (*CR* < 0.1) could be too strict to compare the increasing number of elements and the size of comparison matrix [34]. Therefore, it is possible to conditionally increase the *CR* reference value (e.g., *CR* < 0.2) depending on the research topic [30,31,35]. This research also set a low value (*CR* < 0.2) to consider the issues of the heterogeneity of the students’ answers in online surveys and non-face-to-face semesters due to the control/prevention of the COVID-19 pandemic.

Approval from the research committee is not required for this study, as it falls under the category where the research subjects are not personally defined and the study does not involve the collection of sensitive information, as specified by Article 23 of the Personal Information Protection Act in the Republic of Korea [36,37]. Before implementing this questionnaire, this study repeatedly noted that the survey observed the relevant laws/rules regarding information/privacy protection (e.g., Articles 15 and 17 in the Personal Information Protection Act of the Republic of Korea; [36,37]), and it was performed with informed consent from its questionnaire/survey participants (see Table A1 in the Appendix A).

## 4. Sample Data

### Participants, Data Sources, and Variables

This research conducted surveys of AHP (all 41 questions including demographics and 33 AHP questions in Table A1 and Figure 2) and other demographic questions (Table 2) on 113 students majoring in business administration, engineering, and other fields at a national university in Gangwon State. The research obtained 93 respondents (response rate: 82.3%) and excluded pupils who did not complete the survey. The study implemented a Google-formatted questionnaire because of the Korean government’s social distancing policies.

**Figure 7:**
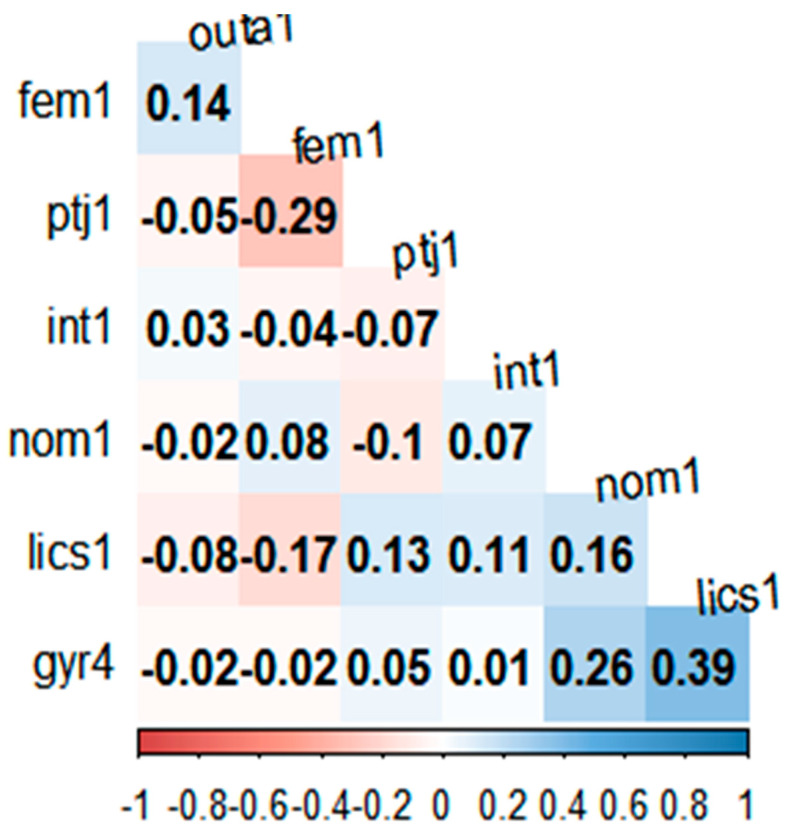
Correlation table between demographic and job-relevant factors. Notes. All the variables in the figures are dummy factors. fem1: female; ptj1: students experiencing a part-time job more than once. int1: students experiencing internships more than once; nom1: students majoring in topics other than management; lics1: students acquiring licenses more than once; gyr4: whether students were freshmen (=1), sophomores (=2), juniors (=3), or seniors (=4); outa1: students experiencing extracurricular activities more than once.

The main characteristics of the respondents (Table 2) show that most had no internship experience (95%), and more than half of the students had no extracurricular activities or internship experience (66.7%, 62.4%). They also had some tendencies to plan to enter private and public companies (85%). The correlation between these demographic variables (Figure 7) suggests that the overall level of correlation could be low (i.e., [−0.29, 0.39]).

## 5. Results

This study presents the results of the pairwise comparison answered by respondents who passed the standard value of the consistency ratio (*CR* < 0.2). This research offers additional analyses with various conditions of job preparation. The research utilized the R software (Version 4.1.1) among software packages (e.g., R, Super Decision, and Expert Choice). The study adopted Yoon and Choi [31]’s analytical flow and R codes (Figure 4).

The main result of the AHP analysis (Table 3) shows that students evaluated performance (54.93%), importance (23.29%), and need for external help (21.78%) highly in the three evaluation criteria for job-preparation activities. They rated participating in extracurricular activities (30.52%), undertaking internships or job-related experience (23.01%), obtaining employment-related qualifications/licenses (21.30%), and achieving high credits in their major courses (16.53%), while networking with university/alumni (8.64%) was underrated. Then, participating in extracurricular activities was highly evaluated, and internship/job experience was high in the criteria of the need for external help. Acquiring employment-related qualifications/licenses was highly valued in terms of performance and importance, while networking activities were rated lowest across the criteria.

Furthermore, as the result of performing sub-analysis according to the respondents’ employment-related factors (Table 4), performance/achievement was the most valuable ([44.98–56.14%]) and common to all the criteria. In the five alternative evaluations, experiences in performing extracurricular activities and internships were generally highly rated, while networking activity was evaluated as the lowest. This research measured the normalized Herfindahl–Hirschman index (*NHHI* (see the equation in the notes of Table 4 [38])), which can provide the degrees/weights of concentration of certain elements. *NHHI* was relatively high in the criteria ([56.36–63.19]), and importance was crucial in general. When *NHHI* was somewhat low in the alternatives ([27.44–31.89]), extracurricular activities were highly evaluated.

**Figure 8:**
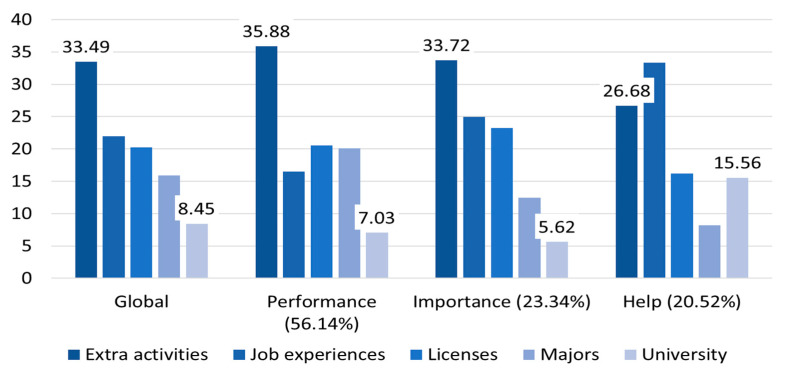
The AHP results without experience in undertaking internships. Notes. Unit: percentage (%); Global: the weights of global (value) are the weighted sum of the local values (performance, importance, and help). Help: need for external help; Extra activities: extracurricular activities; Major: GPA and/or knowledge of major courses; Networking: networking with alumni/universities.

**Figure 9:**
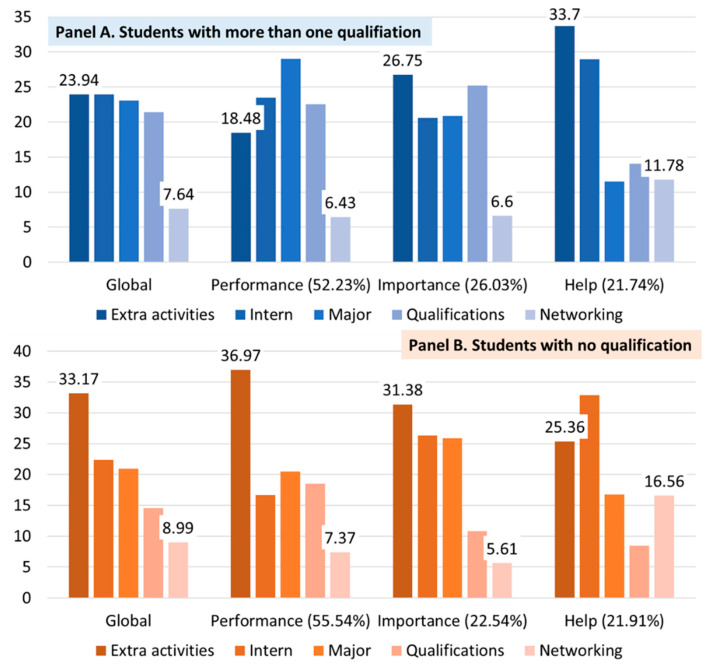
The AHP results with experience in acquiring employment-related qualifications. Notes. Unit: percentage (%); Global: the weights of global (value) are the weighted sum of the local values (performance, importance, and help).

**Figure 10:**
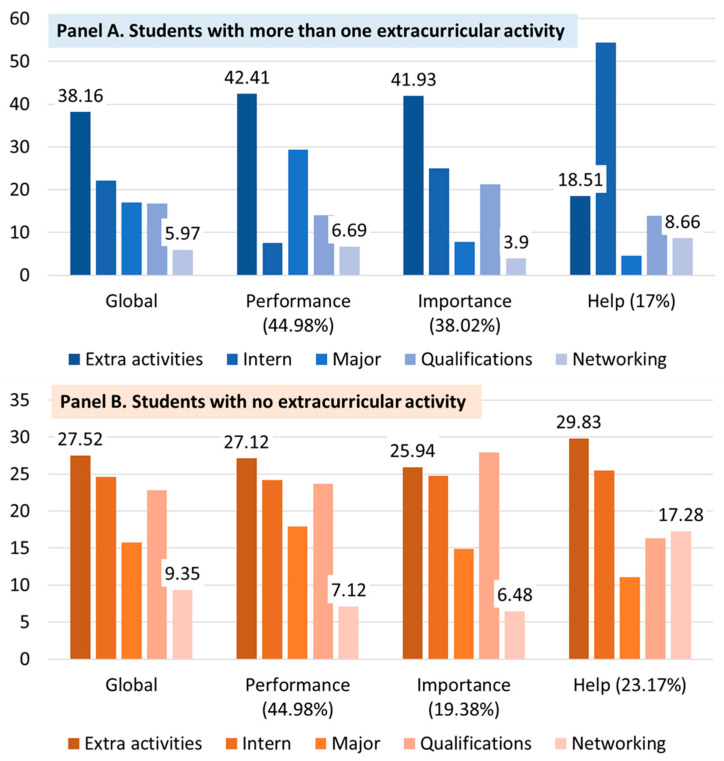
The AHP results with experience in performing extracurricular activities. Notes. Unit: percentage (%); Global: the weights of global (value) are the weighted sum of the local values (performance, importance, and help).

The results of the sub-analysis according to job-related factors (Figure 8, Figure 9 and Figure 10) show that students who do not have experience in internship evaluated the extracurricular activities as the most important both in the global and local results (Figure 8).

Students who had acquired more than one qualification (Panel A in Figure 9) evaluated extracurricular activities and internships the most meaningfully. However, some results differed, depending on the evaluation criteria. The achievement of GPA in major courses was evaluated as meaningful in the achievement criterion. Extracurricular activities were evaluated as significant in terms of both the standard of importance and external help.

Networks with alumni/universities were rated as low in general. Students without qualifications (Panel B in Figure 9) and those with more than one qualification commonly evaluated extracurricular activities as the most important. However, the proportion implementing the activities showed a very large difference in weight from other activities; it was evaluated as the most meaningful in terms of performance and importance. Job-related experience was rated highly based on the criterion of the need for external help.

Lastly, students who performed more than one extracurricular activity (Panel A in Figure 10) evaluated the activity most meaningfully in the overall and local evaluations (i.e., performance and importance). Those who had no experience of the activity (Panel B in Figure 10) also evaluated the activity most meaningfully in the overall and local results (i.e., performance and the need for external help).

## 6. Discussion

### 6.1. Academic Highlights

This research offers the results of the main analysis (Table 3) and subgroup analyses according to various employment-related factors for two research questions (the most meaningful criteria and alternatives in preparing for future careers; Table 4). The common findings across the analyses show that students viewed personal achievement as the most important criterion, and extracurricular activities were the most preferred alternative. The findings could be a robust result that could be derived by sensitivity analysis, which does not cause a large change in ranking for a slight change in the weights of the judgment criteria [24].

This research could be notable in that it applies the logical frameworks of importance, performance, and external support to university students’ career preparation strategies. For instance, in a situation where tourism industries have been severely depressed due to the COVID-19 pandemic, students majoring in this field may face even greater challenges than anticipated due to a scarcity of job opportunities [39]. Therefore, it becomes crucial for undergraduates to develop comprehensive career competencies. This development might involve harnessing their decision-making potential and seeking networking or support from others, such as university mentors or alumni [7].

### 6.2. Practical Implications

This study offers practical guidance for career preparation by outlining undergraduates’ perceptions about essential criteria and preferred strategies for future career planning. According to the AHP results, a sense of achievement emerged as the most important criterion (44.98–57.45% in Panel A of Table 4) across all employment-related conditions. This sense of personal accomplishment seems crucial to students navigating the uncertainties of job hunting, particularly given the downturn brought on by COVID-19. For those with no prior extracurricular experience, the need for external support was rated relatively high (17–23.17% in Table 4). This suggests that gaining career-related experience can help alleviate future career anxieties.

The results also highlight that students value extracurricular activities and internships as key strategies for career preparation (Panel B in Table 4). These activities could suggest some mismatching points between university education and students’ expectations because their education may not have sufficiently provided the activities. Emerging professional paths, such as youth content creation (e.g., YouTubers) and entrepreneurship, may not be adequately covered by traditional university curricula.

These unconventional careers may appeal to Generation Z students, who were born after the mid-1990s [40,41], who may want to learn as they may be dissatisfied with the existing curriculum. Medical departments/colleges have offered several field exercises or internships to students as basic requirements for their graduation, and these students consider their major a stable source of professional vocation [4]. Therefore, business/management schools may need to innovate and diversify their curriculum to include more practical education instead of instructor-initiated traditional capstone designs and team-teaching. For example, it could be useful for higher education to establish some concrete frameworks or roadmaps about career development based on students’ opinion checkup such as representative pathway, work and lifestyle (freelancer, contractor, etc.), geographical positioning (local, national, global, etc.), path dependent on environments, or motivation in sport management [42]. Several demographic factors (e.g., gender [42], age, social background of pupils) could also be influential to design their career management.

Interestingly, students rated networking with alumni and their university as the least valuable activity (Panel B in Table 4). This undervalued networking may be the outcome of their university lives, during which most of them have taken online courses and have not experienced offline interactions with students/acquaintances in their campus for more than two years due to COVID-19 [2].

Lastly, sample-specific backgrounds may offer contextual reasons as to why many opportunities for jobs have been concentrated in Seoul (the capital of Korea) and why young people have been leaving for Seoul and its neighboring areas [43]. Students’ low rating of university reputation could reflect the university’s remote location relative to Seoul. In such circumstances, weak social ties between people might prove useful in providing job information or assistance [44,45]. Therefore, universities and employment-related organizations need to design and implement realistic strategies to motivate and collaborate with students, alumni, and mentors to foster mutual development.

## 7. Conclusions

This study examines the key criteria and activities that Generation Z undergraduates prioritize when preparing for employment in the post-COVID-19 era. The AHP analysis suggests that students consider personal achievements and participation in extracurricular activities as the most crucial elements for career readiness. These findings suggest that accomplishments significantly contribute to boosting students’ confidence amidst the uncertainties of job hunting. Therefore, higher education institutions may need to innovate their educational systems and enhance their learning efficacy, possibly by offering a wider array of extracurricular activities and internships.

To strengthen and generalize these findings, future studies should be undertaken. In particular, future research should use larger, more diverse datasets, such as panel and triangulated/heterogeneous data, instead of relying on self-reported small samples. This would allow for more robust and reliable findings. Further, comparative analyses with different regions, majors, and nations should be designed and conducted to provide more comprehensive insights. As the data become more complex, alternative methodologies could be appropriate, such as ANP, which does not assume independence between elements [23], Fuzzy AHP and TOPIS [25,32,46,47], and MACBETH analyzing the pairwise comparison of measurement/dataset of the interval scale [24]. Finally, both universities and students need to cultivate adaptable, cutting-edge learning skills in the face of the rapidly emerging of hyperscale artificial intelligence such as ChatGPT and Bard. Such technologies carry the potential to revolutionize education, with the public increasingly expecting their application in tutoring. At the same time, concerns regarding their potential misuse as a cheating aid also need to be addressed [48,49].

## Figures and Tables

**Table 1:** Meanings and references of criteria and alternative activities about job preparation.

Elements		Meaning	References
Criteria for judgment	(Relative) importance	The degree of relative influence or importance when I identify/recognize the preparation factors necessary for future employment and determining priorities of the factors	- Importance–performance Analysis (IPA): Azzopardi and Nash [11], Phadermrod, Crowder [12]; - Concern in career adaptability: Akkermans, Paradniké [7], Negru-Subtirica, and Pop [10]
	Performance/Achievement	The feeling of achievement or degree of performance when I identify/recognize the preparation factors necessary for future employment and determining priorities of the factors	- Importance–performance Analysis (IPA): Azzopardi and Nash [11], Phadermrod, Crowder [12]; - Confidence in career adaptability: Akkermans, Paradniké [7], Negru-Subtirica, and Pop [10]
	The need for external help	The degree to which I need help or support of others (mentor) when I identify/recognize the preparation factors necessary for future employment and determining priorities of the factors	Control in career adaptability: Akkermans, Paradniké [7], Negru-Subtirica, and Pop [10]
Alternative activities	Understanding my major courses	To increase my knowledge, GPA, or expertise in my major	Grade point average (GPA): Kim, Min [1], Akkermans, Paradniké [7]
	Networking activities with alumni/universities	To cooperate with my university and alumni for plausible employment	- Success of my school: Önder, Önder [6]; - Alumni’s reputation or image: Kang and Park [20]
	Acquiring qualifications for employment	To obtain the required qualifications for future employment (e.g., TOEIC, OPIc)	Kim, Min [1]
	Performing interns or job-relevant activities	To undertake an internship or work experience in the field or with the company/organization I want to work in	Intern: Kim, Min [1], Pan, Guan [21]
	Participating in extracurricular activities	To take part in competition fairs/exhibitions that could be necessary or helpful in the field or the company/organization I want to work in	Kim, Min [1]

Notes. TOEIC: Test of English for International Communication; OPIc: Oral Proficiency Interview Computer.

**Table 2:** Descriptive statistics of demographic and job-relevant factors.

Demographic Factors	Features	
Gender	Male: 48 persons (51.6%)	Female: 45 persons (48.4%)
Year of grades	Freshman: 26 persons (28%) Junior: 37 persons (39.8%)	Sophomore: 19 persons (20.4%) Senior: 11 persons (11.8%)
Major	Non-management: 83 persons (89.2%)	Management: 10 persons (10.8%)
Experiences on performing intern or other job-related activities	None: 88 persons (94.6%) Twice/More than three times: none	Once: 5 persons (5.4%)
Experiences on participating in extracurricular activities (e.g., competition exhibitions/fairs)	None: 62 persons (66.7%) Twice: 6 persons (6.5%)	Once: 16 persons (17.2%) More than three times: 9 persons (9.7%)
Experiences on acquiring certificates/licenses for jobs (e.g., TOEIC, OPIc)	None: 58 persons (62.4%) Twice: 6 persons (6.5%)	Once: 26 persons (28%) More than three times: 3 persons (3.2%)
Experiences on doing part-time works	None: 10 persons (10.8%) Twice: 13 persons (14%)	Once: 25 persons (26.9%) More than three times: 45 persons (48.4%)
Preferred fields of workplaces for their jobs	Companies: 79 persons (84.9%) Starting a business or participating in a family business: 11 persons (11.8%)	Public institutions: 18 persons (19.4%) Others: 3 persons (3.2%)

Notes. TOEIC: Test of English for International Communication; OPIc: Oral Proficiency Interview Computer.

**Table 3:** Main results by AHP analysis.

Alternatives	Global Evaluations		Local Evaluations					
			Performance (WE: 54.93%)		Relative Importance (WE: 23.29%)		Needs for Help (WE: 21.78%)	
	RA	WE (%)	RA	WE (%)	RA	WE (%)	RA	WE (%)
Extra activities	1	30.52	1	31.80	1	30.27	2	27.57
Job experiences	2	23.01	4	18.48	3	25.14	1	32.15
Licenses	3	21.30	2	21.40	2	25.91	3	16.11
Majors	4	16.53	3	21.06	4	12.81	5	9.06
University	5	8.64	5	7.26	5	5.86	4	15.11
Total	-	100	-	100	-	100	-	100

Notes. The gray areas represent the two highest weights of the elements in each column. RA: rankings; WE: weights; Major: GPA and/or knowledge of major courses; Networking: networking with alumni/universities; -: Not available. The weights were rounded to three decimal places.

**Table 4:** Results of sub-analysis with different features of job preparation.

Conditions	All (Table 3)	Intern	Licenses		Extra-Activities	
		None	More than Once	None	More than Once	None
**Panel A. Weights of three criteria (Sum of each column: 100%)**						
Performances	54.93	56.14	52.23	55.54	44.98	57.45
Relative importance	23.29	23.34	26.03	22.54	38.02	19.38
Needs for help	21.78	20.52	21.74	21.91	17.00	23.17
*NHHI*	60.51	61.76	58.17	61.09	56.36	63.19
**Panel B. Weights of five alternatives (Sum of each column: 100%)**						
Extra activities	30.52	33.49	23.94	33.17	38.16	27.52
Job experiences	23.01	21.93	23.92	22.40	22.16	24.60
Licenses	21.30	20.26	21.41	20.90	16.75	22.81
Majors	16.53	15.87	23.10	14.55	16.96	15.73
University	8.64	8.45	7.64	8.99	5.97	9.35
*NHHI*	28.28	29.20	27.44	29.14	31.89	27.72

Notes. The gray areas are the two highest weights of elements in each column. Values of weights are rounded to three decimal places. Most students did not have experience of internships. Major: GPA and/or knowledge of major courses; Networking: networking with alumni/universities; *NHHI*: normalized Herfindahl–Hirschman index. *NHHI* =(D−1/n)(D−1/n). $D=\overset{n}{\underset{i=1}{\sum }}s_{i}^{2}.$ ($s_{i}^{}$: the squared value of *i*’s proportion in the total; *n:* the number of elements in a layer.) When the *NHHI* is close to zero, the proportion portfolio of the elements appears to be very balanced (please see Lee, Kang [38]).

## Data Availability

The data presented in this study are available on request from the corresponding author.

## Appendix A

**Table A1:** Forty-one survey questions in this research (Panel A, B, C1, C2, C3, and D).

**Panel A. Announcement on the Privacy Laws and the Informed Consent**								
1. This survey complies with all relevant laws/rules concerning information/privacy protection, specifically Article of 15 and 17 in the Personal Information Protection Act of Republic of Korea. 2. The participants’ information will be used exclusively for research purposes and to improve the quality of relevant courses. 3. All information collected during this survey will be kept strictly confidential and will be deleted immediately after we have completed our analysis. 4. If you do not want to participate in this survey, you can stop answering the survey without completion.								
**Panel B. Three questions on comparative priorities between decision criteria**								
In this survey, we would like to ask you to compare **three aspects: relative importance, performance (or achievement), and the need for external help** for your priority to prepare for future jobs and/or career development.								
1. When it comes to preparing for future jobs or career development, which of the following options do you prioritize more: **relative importance** or **performance?** Please select one of the following answers (A to I) based on your preference.								
A	B	C	D	E	F	G	H	I
I prefer importance.	-	-	-	I prefer them equally.	-	-	-	I prefer performance.
2. When it comes to preparing for future jobs or career development, which of the following options do you prioritize more: **relative importance** or the **need for external help?** Please select one of the following answers (A to I) based on your preference.								
A	B	C	D	E	F	G	H	I
I prefer importance.	-	-	-	I prefer them equally.	-	-	-	I prefer the need for external help.
3. When it comes to preparing for future jobs or career development, which of the following options do you prioritize more: **performance** or the **need for external help?** Please select one of the following answers (A to I) based on your preference.								
A	B	C	D	E	F	G	H	I
I prefer performance.	-	-	-	I prefer them equally.	-	-	-	I prefer the need for external help.
**Panel C1. Ten questions on comparative priorities between alternative activities based on the criterion of importance**								
We would like to ask you to participate in this survey by comparing five alternative activities based on their importance for preparing for future jobs or career development. The activities we are interested in are as follows: 1. GPA in major courses 2. Networking with alumni/university 3. Job-related qualifications 4. Internship/job experiences 5. Extracurricular activities (such as competition exhibitions/fairs)								
4. When it comes to preparing for future jobs or career development, which of the following options do you prioritize more: **GPA in major courses** or **networking with alumni/university?** Please select one of the following answers (A to I) based on the criterion of **importance.**								
A	B	C	D	E	F	G	H	I
I prefer GPA in major courses.	-	-	-	I prefer them equally.	-	-	-	I prefer networking with alumni/university.
5. When it comes to preparing for future jobs or career development, which of the following options do you prioritize more: **GPA in major courses** or **job-related qualifications?** Please select one of the following answers (from A to I) based on the criterion of **importance.**								
A	B	C	D	E	F	G	H	I
I prefer GPA in major courses.	-	-	-	I prefer them equally.	-	-	-	I prefer job-related qualifications.
6. When it comes to preparing for future jobs or career development, which of the following options do you prioritize more: **GPA in major courses** or **intern/job experiences?** Please select one of the following answers (from A to I) based on the criterion of **importance.**								
A	B	C	D	E	F	G	H	I
I prefer GPA in major courses.	-	-	-	I prefer them equally.	-	-	-	I prefer intern/job experiences.
7. When it comes to preparing for future jobs or career development, which of the following options do you prioritize more: **GPA in major courses** or **extracurricular activities?** Please select one of the following answers (A to I) based on the criterion of **importance.**								
A	B	C	D	E	F	G	H	I
I prefer GPA in major courses.	-	-	-	I prefer them equally.	-	-	-	I prefer extracurricular activities.
8. When it comes to preparing for future jobs or career development, which of the following options do you prioritize more: **networking with alumni/university** or **job-related qualifications?** Please select one of the following answers (A to I) based on the criterion of **importance.**								
A	B	C	D	E	F	G	H	I
I prefer networking with alumni/university.	-	-	-	I prefer them equally.	-	-	-	I prefer job-related qualifications.
9. When it comes to preparing for future jobs or career development, which of the following options do you prioritize more: **networking with alumni/university** or **intern/job experiences?** Please select one of the following answers (A to I) based on the criterion of **importance.**								
A	B	C	D	E	F	G	H	I
I prefer networking with alumni/university.	-	-	-	I prefer them equally.	-	-	-	I prefer intern/job experiences.
10. When it comes to preparing for future jobs or career development, which of the following options do you prioritize more: **networking with alumni/university** or **extracurricular activities?** Please select one of the following answers (A to I) based on the criterion of **importance.**								
A	B	C	D	E	F	G	H	I
I prefer networking with alumni/university.	-	-	-	I prefer them equally.	-	-	-	I prefer extracurricular activities.
11. When it comes to preparing for future jobs or career development, which of the following options do you prioritize more: **job-related qualifications** or **intern/job experiences?** Please select one of the following answers (A to I) based on the criterion of **importance.**								
A	B	C	D	E	F	G	H	I
I prefer job-related qualifications.	-	-	-	I prefer them equally.	-	-	-	I prefer intern/job experiences.
12. When it comes to preparing for future jobs or career development, which of the following options do you prioritize more: **job-related qualifications** or **extracurricular activities?** Please select one of the following answers (A to I) based on the criterion of **importance.**								
A	B	C	D	E	F	G	H	I
I prefer job-related qualifications.	-	-	-	I prefer them equally.	-	-	-	I prefer extracurricular activities.
13. When it comes to preparing for future jobs or career development, which of the following options do you prioritize more: **intern/job experiences** or **extracurricular activities?** Please select one of the following answers (A to I) based on the criterion of **importance.**								
A	B	C	D	E	F	G	H	I
I prefer intern/job experiences.	-	-	-	I prefer them equally.	-	-	-	I prefer extracurricular activities.
**Panel C2. Ten questions on comparative priorities between alternative activities based on the criterion of performance**								
We would like to ask you to participate in this survey by comparing five alternative activities based on their performance for preparing for future jobs or career development. The activities we are interested in are as follows: 1. GPA in major courses 2. Networking with alumni/university 3. Job-related qualifications 4. Internship/job experiences 5. Extracurricular activities (such as competition exhibitions/fairs)								
14. When it comes to preparing for future jobs or career development, which of the following options do you prioritize more: **GPA in major courses** or **networking with alumni/university?** Please select one of the following answers (A to I) based on the criterion of **performance.**								
A	B	C	D	E	F	G	H	I
I prefer GPA in major courses.	-	-	-	I prefer them equally.	-	-	-	I prefer networking with alumni/university.
15. When it comes to preparing for future jobs or career development, which of the following options do you prioritize more: **GPA in major courses** or **job-related qualifications?** Please select one of the following answers (A to I) based on the criterion of **performance.**								
A	B	C	D	E	F	G	H	I
I prefer GPA in major courses.	-	-	-	I prefer them equally.	-	-	-	I prefer job-related qualifications.
16. When it comes to preparing for future jobs or career development, which of the following options do you prioritize more: **GPA in major courses** or **intern/job experiences?** Please select one of the following answers (A to I) based on the criterion of **performance.**								
A	B	C	D	E	F	G	H	I
I prefer GPA in major courses.	-	-	-	I prefer them equally.	-	-	-	I prefer intern/job experiences.
17. When it comes to preparing for future jobs or career development, which of the following options do you prioritize more: **GPA in major courses** or **extracurricular activities?** Please select one of the following answers (A to I) based on the criterion of **performance.**								
A	B	C	D	E	F	G	H	I
I prefer GPA in major courses.	-	-	-	I prefer them equally.	-	-	-	I prefer extracurricular activities.
18. When it comes to preparing for future jobs or career development, which of the following options do you prioritize more: **networking with alumni/university** or **job-related qualifications?** Please select one of the following answers (A to I) based on the criterion of **performance.**								
A	B	C	D	E	F	G	H	I
I prefer networking with alumni/university.	-	-	-	I prefer them equally.	-	-	-	I prefer job-related qualifications.
19. When it comes to preparing for future jobs or career development, which of the following options do you prioritize more: **networking with alumni/university** or **intern/job experiences?** Please select one of the following answers (A to I) based on the criterion of **performance.**								
A	B	C	D	E	F	G	H	I
I prefer networking with alumni/university.	-	-	-	I prefer them equally.	-	-	-	I prefer intern/job experiences.
20. Please compare between **networking with alumni/university** or **extracurricular activities** and check one of the following answers (A to I) based on the criterion of **performance.**								
A	B	C	D	E	F	G	H	I
I prefer networking with alumni/university.	-	-	-	I prefer them equally.	-	-	-	I prefer extracurricular activities.
21. When it comes to preparing for future jobs or career development, which of the following options do you prioritize more: **job-related qualifications** or **intern/job experiences?** Please select one of the following answers (A to I) based on the criterion of **performance.**								
A	B	C	D	E	F	G	H	I
I prefer job-related qualifications.	-	-	-	I prefer them equally.	-	-	-	I prefer intern/job experiences.
22. When it comes to preparing for future jobs or career development, which of the following options do you prioritize more: **job-related qualifications** or **extracurricular activities?** Please select one of the following answers (A to I) based on the criterion of **performance.**								
A	B	C	D	E	F	G	H	I
I prefer job-related qualifications.	-	-	-	I prefer them equally.	-	-	-	I prefer extracurricular activities.
23. When it comes to preparing for future jobs or career development, which of the following options do you prioritize more: **intern/job experiences** or **extracurricular activities?** Please select one of the following answers (A to I) based on the criterion of **performance.**								
A	B	C	D	E	F	G	H	I
I prefer intern/job experiences.	-	-	-	I prefer them equally.	-	-	-	I prefer extracurricular activities.
**Panel C3. Ten questions on comparative priorities between alternative activities based on the criterion of need for external help**								
We would like to ask you to participate in this survey by comparing five alternative activities based on their performance for preparing for future jobs or career development. The activities we are interested in are as follows: 1. GPA in major courses 2. Networking with alumni/university 3. Job-related qualifications 4. Internship/job experiences 5. Extracurricular activities (such as competition exhibitions/fairs)								
24. When it comes to preparing for future jobs or career development, which of the following options do you prioritize more: **GPA in major courses** or **networking with alumni/university?** Please select one of the following answers (A to I) based on the criterion of the **need for external help.**								
A	B	C	D	E	F	G	H	I
I prefer GPA in major courses.	-	-	-	I prefer them equally.	-	-	-	I prefer networking with alumni/university.
25. When it comes to preparing for future jobs or career development, which of the following options do you prioritize more: **GPA in major courses** or **job-related qualifications?** Please select one of the following answers (A to I) based on the criterion of the **need for external help.**								
A	B	C	D	E	F	G	H	I
I prefer GPA in major courses.	-	-	-	I prefer them equally.	-	-	-	I prefer job-related qualifications.
26. When it comes to preparing for future jobs or career development, which of the following options do you prioritize more: **GPA in major courses** or **intern/job experiences?** Please select one of the following answers (A to I) based on the criterion of the **need for external help.**								
A	B	C	D	E	F	G	H	I
I prefer GPA in major courses.	-	-	-	I prefer them equally.	-	-	-	I prefer intern/job experiences.
27. When it comes to preparing for future jobs or career development, which of the following options do you prioritize more: **GPA in major courses** or **extracurricular activities?** Please select one of the following answers (A to I) based on the criterion of the **need for external help.**								
A	B	C	D	E	F	G	H	I
I prefer GPA in major courses.	-	-	-	I prefer them equally.	-	-	-	I prefer extracurricular activities.
28. When it comes to preparing for future jobs or career development, which of the following options do you prioritize more: **networking with alumni/university** or **job-related qualifications?** Please select one of the following answers (A to I) based on the criterion of the **need for external help.**								
A	B	C	D	E	F	G	H	I
I prefer networking with alumni/university.	-	-	-	I prefer them equally.	-	-	-	I prefer job-related qualifications.
29. When it comes to preparing for future jobs or career development, which of the following options do you prioritize more: **networking with alumni/university** or **intern/job experiences?** Please select one of the following answers (A to I) based on the criterion of **need for external help.**								
A	B	C	D	E	F	G	H	I
I prefer networking with alumni/university.	-	-	-	I prefer them equally.	-	-	-	I prefer intern/job experiences.
30. When it comes to preparing for future jobs or career development, which of the following options do you prioritize more: **networking with alumni/university** or **extracurricular activities?** Please select one of the following answers (A to I) based on the criterion of the **need for external help.**								
A	B	C	D	E	F	G	H	I
I prefer networking with alumni/university.	-	-	-	I prefer them equally.	-	-	-	I prefer extracurricular activities.
31. When it comes to preparing for future jobs or career development, which of the following options do you prioritize more: **job-related qualifications** or **intern/job experiences?** Please select one of the following answers (A to I) based on the criterion of the **need for external help.**								
A	B	C	D	E	F	G	H	I
I prefer job-related qualifications.	-	-	-	I prefer them equally.	-	-	-	I prefer intern/job experiences.
32. When it comes to preparing for future jobs or career development, which of the following options do you prioritize more: **job-related qualifications** or **extracurricular activities?** Please select one of the following answers (A to I) based on the criterion of **need for external help.**								
A	B	C	D	E	F	G	H	I
I prefer job-related qualifications.	-	-	-	I prefer them equally.	-	-	-	I prefer extracurricular activities.
33. When it comes to preparing for future jobs or career development, which of the following options do you prioritize more: **intern/job experiences** or **extracurricular activities?** Please select one of the following answers (A to I) based on the criterion of the **need for external help.**								
A	B	C	D	E	F	G	H	I
I prefer intern/job experiences.	-	-	-	I prefer them equally.	-	-	-	I prefer extracurricular activities.
**Panel D. Eight questions on demographic variables of participants**								
We would like to ask you to answer the following questions about your demographic information to better understand our survey participants.								
34. What is your gender? Please select one of the following options: 1. Male. 2. Female.								
35. What year are you in? Please select one of the following options: 1. Freshman. 2. Sophomore. 3. Junior. 4. Senior.								
36. What is your first major? Please select one of the following options: 1. Management. 2. Other.								
37. How many times have you done an internship or other job-related activity? Please select one of the following options: 1. None. 2. Once. 3. More than twice.								
38. How many times have you participated in extracurricular activities? Please select one of the following options: 1. None. 2. Once. 3. Twice. 4. More than three times.								
39. How many times have you acquired certificates/licenses for jobs (e.g., TOEIC, OPIc)? Please select one of the following options: 1. None. 2. Once. 3. Twice. 4. More than three times.								
40. How many times have you done part-time work? Please select one of the following options: 1. None. 2. Once. 3. Twice. 4. More than three times.								
41. Where do you prefer to work in the future? Please select one of the following options: 1. Companies. 2. Public institutions. 3. Starting a business (or participating in a family business). 4. Other (Please specify).								
This is the end of our survey. Thank you for your participation. If you have any questions or concerns, please do not hesitate to contact us.								
